# Enhanced Antiproliferative Effect of Carboplatin in Cervical Cancer Cells Utilizing Folate-Grafted Polymeric Nanoparticles

**DOI:** 10.1186/s11671-015-1162-2

**Published:** 2015-11-25

**Authors:** Jing Ji, Ping Zuo, Yue-Ling Wang

**Affiliations:** Department of Gynecology & Obstetrics, The First Affiliated Hospital of Xi’An, Jiaotong University, No. 277, Yanta Xi Road, Xian, Shanxi 710061 China

**Keywords:** Cervical cancers, Folate, Chitosan, PLGA nanoparticles, Apoptosis, Targeting

## Abstract

Carboplatin (CRB) possesses superior anticancer effect in cervical cancer cells with lower incidence of side effects compared to that of cisplatin. However, CRB suffers from severe side effects due to undesirable tissue distributions which contribute to the low therapeutic efficacy. Here, we report a unique folic acid-conjugated chitosan-coated poly(d-l-lactideco-glycolide) (PLGA) nanoparticles (FPCC) prepared for the selective delivery of carboplatin to the cervical cancer cells. The particles were nanosized and spherical shaped with size less than <200 nm. The presence of protective chitosan layer controlled the overall release rate of CRB from chitosan-coated PLGA nanoparticles (PCC) and FPCC. FPCC displayed a higher cellular uptake capacity in HeLa cells than compared to non-targeted nanoparticles. Selective uptake of FPCC was due to an interaction of folic acid (FA) with the folate receptors alpha (FRs-α) which is overexpressed on the HeLa and promoted active targeting. These results indicated that FPCC had a specific affinity for the cancerous, HeLa cells owing to ligand-receptor (FA-FR-α) recognition. Consistently, FPCC showed superior cytotoxic effect than any other formulations. The IC_50_ (concentration of the drug required to kill 50 % of the cells) value of FPCC was 0.65 μg/ml while it was 1.08, 1.56, and 2.35 μg/ml for PCC, PLGA NP, and free CRB, respectively. Consistent with the cytotoxicity assay, FPCC induced higher fraction of early as well as late apoptosis cells. Especially, FPCC induced nearly 45 % of early apoptosis cells and more than 35 % in late apoptosis. Therefore, we propose that folate-conjugated nanoparticles might have potential applications in cervical cancer therapy.

## Background

Cervical cancer is the third most common cancer in women and caused by human papillomavirus (HPV) [1]. Although advanced cancer screening and vaccine reduced the mortality rate of patients due to cervical cancers in developed countries, it is still the leading cause of cancer-related death in developing countries like China [2, 3]. There are many treatment options for cervical cancers including surgery, chemotherapy, and radiotherapy; however, none was effective in completing curing the cancers and often recurrence was seen. Recently, two prophylactic HPV vaccines (Gardasil and Cervarix) were approved for the HPV-associated diseases. However, these vaccines are effective only in adolescents with no history of previous HPV infection and have not shown any therapeutic effects against current HPV infections or cervical cancers [4, 5]. Therefore, there is an urgent need to design new approaches and methods to treat cervical cancers.

In this regard, platinum-based drugs (carboplatin and cisplatin) are indicated in the treatment of multiple cancers including cervical cancers [6]. Carboplatin (CRB) (cis-diamine (1,1-cyclobutanedicarboxylato)-platinum (II)), a cisplatin analog, is reported to possess superior anticancer effect in cancer cells and lower incidence of side effects compared to that of cisplatin [7, 8]. Despite the potential anticancer effect, CRB suffers from severe myelosuppression, thrombocytopenia, and leucopenia (in older patients). Furthermore, lower cellular uptake of CRB in cancer cells also contributes to the low therapeutic efficacy [9, 10]. Therefore, it is important to protect the CRB in the blood circulation, and exposure to cancer cells is prolonged to achieve maximum cytotoxic activity. In order to overcome all the drawbacks, we have designed a targeted delivery system which is specific to the cervical cancer cell and thereby increase the therapeutic effect and decrease the side effects.

Ligand conjugation to the carrier system could enhance the specific delivery of drug to the cancer cells. Generally, functionalized nanosystem could target the molecular receptors which are overexpressed in the tumor cells and release its active payload [11]. Recently, polymeric nanoparticles ranging between 100 and 200 nm have gained significant attention due to its high drug-loading capacity, non-immunogenicity, and biodegradability [12, 13]. In the present study, poly(d-l-lactideco-glycolide) (PLGA) polymer has been selected to incorporate CRB. The excellent features of PLGA NP include prolonged systemic circulation, effective intracellular delivery, excellent biocompatibility, and biodegradability. However, drug leakage in the blood circulation and negative charge on the particle surface might diminish the activity of incorporate drug [14–16]. Therefore, chitosan (CS) which is a naturally occurring biodegradable polymer was coated on the PLGA surface [17]. The high zeta potential of chitosan (positively charged) enables the easy surface modification of PLGA NP via electrostatic interactions [18]. The surface modification with chitosan presents many advantages including protection of active drug in the systemic circulation, as a stealth protective layer, and increases the intracellular uptake (due to the interaction of positively charged particle with negatively charged cell membrane) [19–21].

Among various low and high molecular weight ligands (folate, mannose, transferrin), folic acid which is overexpressed in cervical cancer cells (HeLa), was selected to deliver the CRB to the tumor region [22]. Functional folate receptors alpha (FRs-α) are highly overexpressed in cervical cancer cells [23]. FR-α is a glycosyl phosphatidyl inositol (GPI)-linked protein which is present in the apical region of normal cells which is not exposed to the blood circulation, whereas in case of cancer cells, it is exposed to the blood circulation and therefore, folate-conjugated nanoparticles internalized with high affinity (receptor-mediated endocytosis) [24, 25]. There are several reports regarding the intracellular delivery of folate-conjugated nanopharmaceuticals in FR-α overexpressed cancer cells [26].

**Fig. 1:**
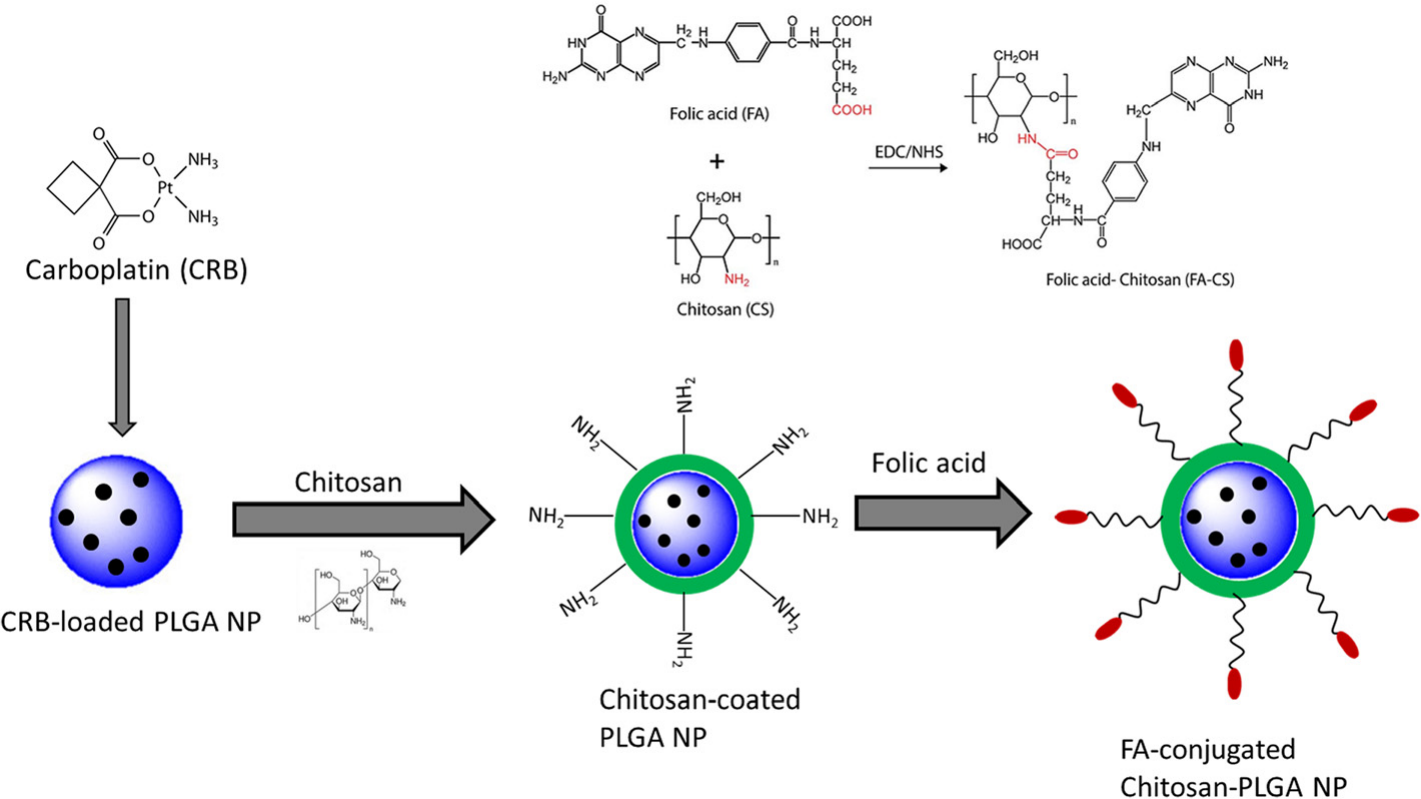
Schematic presentation of preparation of folic acid-conjugated chitosan-coated PLGA nanoparticles. Chitosan-folic acid conjugation was separately depicted

Carboplatin (CRB) is an excellent drug to treat cervical cancers; however, it leads to severe side effects mainly due to the irrational and undesirable biodistribution. Additionally, abnormal extracellular matrix offers accelerated frictional resistance of the anticancer drug penetration. Therefore, active targeting of CRB to the cancer cells increase the concentration of drug on the tumor site while it minimized the associated side effects. In the present study, therefore, we have designed a unique folic acid-conjugated chitosan-coated PLGA nanoparticle (FPCC). The high zeta potential of chitosan (positively charged) enables the easy surface modification of PLGA NP via electrostatic interactions (Fig. 1). FA over the surface of nanoparticles remarkably influenced the therapeutic efficacy of encased cytotoxic drug.

Therefore, in the present investigation, folate-grafted chitosan/PLGA nanoparticle was prepared to deliver carboplatin to the FR-α overexpressed HeLa cervical cancer cells. The physicochemical characteristics of optimized nanoformulations were studied by dynamic light scattering (DLS) technique. In vitro drug release profile was investigated for different formulations. Cellular uptake efficiency of targeted and non-targeted nanoparticles was investigated to prove the targetability of folate-grafted carrier. Consistently, cytotoxicity assay and apoptosis analysis were carried out to show the superior anticancer effect of folate-grafted nanocarrier.

## Methods

### Materials

Carboplatin, folic acid, PLGA 50:50, MW 40,000–75,000 Da, dexamethasone (DXM) ≥98 %, chitosan (CS) 75–85 % deacetylated, average MW 50,000 Da, and all other chemicals were purchased from Sigma-Aldrich, China.

### Preparation of Folic Acid-Conjugated Chitosan-Coated PLGA Nanoparticles

One hundred milligrams of PLGA and 25 mg of CRB were dissolved in 5 ml of dimethylsulfoxide (DMSO). The solution was added dropwise into 25 ml of a selected non-solvent of the polymer (H_2_O). The solution was stirred for 3 h until all the organic solvent evaporated. Subsequently, titrated volume of 1 % chitosan solution was added to the PLGA solution under constant stirring at room temperature. The suspension was then centrifuged at 10,000 rpm for 30 min at 4 °C, and the supernatant was removed. The pellet was then washed twice with deionized water and recovered by centrifugation.

Separately, chitosan-folic acid (CH-FA) was synthesized by chemical conjugation. Briefly, 10 mg of folic acid (FA) was added to a mixture of 1-(3-dimethylaminopropyl)-3-ethyl carbodiimide hydrochloride (EDAC.HCl) and n-hydroxy succinimide (NHS) (10:25 mg) prepared in 10 mL of DMSO. The activation was carried out under inert atmosphere for 30 min. The activated FA solution was then added dropwise to the chitosan solution. The product was filtered and dried. The amount of CRB entrapped in the nanoparticle was evaluated by filtration method. The drug-loaded nanoparticle dispersion was filtered, and the supernatant was subject to ICP-MS method to evaluate the entrapment efficiency and loading capacity in the nanoparticles.

### Particle Size and Zeta Potential Analysis

The average particle diameter, particle size distribution, and surface charge were determined using dynamic light scattering (DLS) technique by Zetasizer Nano ZS (Malvern, UK) at 25 °C. The formulations were suitably diluted before the experiment.

### Transmission Electron Microscopy (TEM) Observation

The morphology and size of the particles were observed using transmission electron microscopy (Jeol JEM1200EX, Tokyo, Japan) operated at an accelerated voltage of 80 kV. The samples were negatively stained with 2 % PTA and then placed a drop in the copper grid, air dried, and observed under microscope.

### In Vitro Release Study

CRB-loaded PLGA NP, PCC, and FPCC was dissolved in 2 ml of buffer to form a suspension and then transferred to dialysis membrane (Spectra/Por 6, MWCO 3000, Spectrum Laboratories, Inc., TX, USA). The bag was closed and immersed in 20 ml the release medium and put in an orbital shaker set at 100 rpm at 37 °C. From time to time, 1 ml of the release medium was withdrawn and replaced with equal amount of fresh buffer. The amount of drug released was determined by ICP-MS method.

### Cell Culture

Human cervical carcinoma cell line HeLa cells were cultured in DMEM supplemented with 10 % fetal bovine serum (FBS), 100 U/mL penicillin, and 100 mg/mL streptomycin at 37 °C in a humidified atmosphere containing 5 % carbon dioxide.

### Cellular Uptake Analysis

The cellular uptake of nanoparticles in HeLa cells was determined by fluorescence method. In brief, 2 × 10^4^ cells were seeded in a 96-well plate and incubated overnight. The medium was removed and treated with fresh medium containing PLGA NP, PCC, and FPCC. To study the effect of targeted and non-targeted nanoparticles, coumarin-6 was used as a fluorescent probe. The formulations were incubated for 1, 2, and 4 h and then washed with PBS. Cells were lysed with the help of lysis buffer (1 % Triton-X in 0.2 N NaOH), and the fluorescent intensity of probe was evaluated by means of microplate reader (GENios, Tecan, Switzerland) with excitation wavelength at 430 nm and emission wavelength at 485 nm.

### Cellular Uptake Imaging

The qualitative cellular uptake of nanoparticles in HeLa was evaluated using a confocal laser scanning microscope (Olympus Fluoview FV-1000, Tokyo, Japan). For this purpose, cells were seeded in six-well plate at a seeding density of 2 × 10^5^ cells and incubated for 24 h. The cells were then exposed with the coumarin-6-loaded formulations and incubated for 2 h. The cells were washed three times with cold PBS, fixed by cold methanol for 20 min, and further washed twice with PBS. The cells were then viewed under CLSM microscope.

### Cytotoxicity Assay

The in vitro cytotoxicity assay of free CRB, PLGA NP, PCC, and FPCC was studied in HeLa cancer cells by MTT assay protocol. For this purpose, 1 × 10^4^ cells which were in the logarithmic phase of growth were seeded in each well of 96-well plate. As mentioned above, DMEM supplemented with 10 % FBS and 1 % antibiotic was used as a growth media. When the confluence of cells reaches 85 %, old media was removed and replaced with new media containing individual formulations in a dose-dependent manner and incubated for 24 h. After that, 10 μl of MTT solution (5 mg/ml) was added to each well of 96-well plate and incubated for additional 4 h. The MTT solution was slowly aspired and 100 μl of DMSO was added to dissolve the purple color formazan crystals. The absorbance of formazan was evaluated using Bio-Tek Synergy HT plate reader (Bio-Tek Instruments Inc., USA) at 570 nm.

$$ \mathrm{Cell}\ \mathrm{viability}\left(\%\right)=\frac{\mathrm{Absorbance}\ \mathrm{o}\mathrm{f}\ \mathrm{cells}\ \mathrm{exposed}\ \mathrm{t}\mathrm{o}\ \mathrm{samples}\kern0.5em \times \kern0.5em 100\%}{\mathrm{Absorbance}\ \mathrm{o}\mathrm{f}\ \mathrm{untreated}\ \mathrm{cells}} $$

### Apoptosis Analysis

The apoptosis assay was carried out using a flow cytometer (FACS). Briefly, 5 × 10^5^ cells were seeded in a six-well plate and incubated for 24 h. The cells were then treated with free CRB, PLGA NP, PCC, and FPCC and incubated for additional 24 h. The cells were then harvested with trypsin-EDTA, centrifuged and washed twice with ice-cold PBS. Subsequently, binding buffer (1×) was added to each tube. Then, 100 μl of cell suspension was transferred to FACS tube and treated with 5 μl of annexin and 5 μl of 17-AAD, vortexed, and incubated for 15 min. After mixing, 400 μl of binding buffer was added to each tube and apoptosis was analyzed using flow cytometer (Beckman Coulter, Inc., Fullerton, CA, USA). Here, the total apoptosis includes both early and late apoptosis.

### Statistical Analysis

All data were analyzed by paired *t* test using SPSS 11.0 software. Differences were considered statistically significant at *P* < 0.05.

## Results and Discussion

### Particle Size and Surface Characteristics of Nanoparticles

The mean size of different nanoparticles is presented in Table 1. The average size of PLGA NP was 85 nm while particle size increased to 145 nm for the deposition of chitosan on the surface (chitosan-coated PLGA NP (PCC)). The particle size of folic acid-conjugated PCC NP however increased to 190 nm with a narrow size distribution. The gradual increase in the average particle size was attributed to the deposition of additional materials [27]. Consistently, zeta potential was checked to confirm the formation of nanoparticles. The PLGA has a strong negative charge around −20 mV while it reversed to +26.5 mV for the additional of chitosan indicating a firm deposition of additional mass on the polymer surface. It has been reported that intracellular uptake of nanoparticles in tumor tissue was influenced by the size of nanoparticles [11, 12]. The optimized nanoparticles (FPCC) in the present study are very much suitable for the cancer targeting study. FPCC displayed high entrapment efficiency (EE) of more than 90 % with an effective loading of 11.85 %. It should be noted that conjugation of folic acid did not alter the loading capacity of formulated drug.

**Table 1:** Characterization of drug-loaded nanoparticles

	Size (nm)	Charge (mV)	PDI	EE (%)	DL (%)
PLGA NP	85.5 ± 1.86	−20.5 ± 1.15	0.124	98.2 ± 2.35	16.5 ± 1.84
PCC NP	145.8 ± 2.1	+26.5 ± 1.65	0.168	95.2 ± 3.65	12.56 ± 1.65
FPCC NP	189.5 ± 1.8	+21.6 ± 1.19	0.198	94.6 ± 2.46	11.85 ± 1.95

### Surface Characteristics

**Fig. 2:**
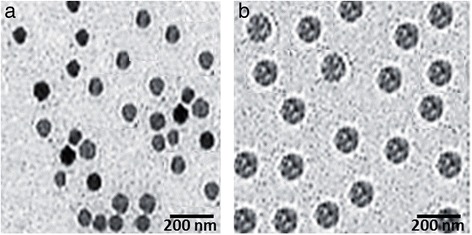
Transmission electron microscope (TEM) imaging of **a** chitosan-coated PLGA nanoparticles and **b** folic acid-conjugated chitosan-coated PLGA nanoparticles

The morphology of PCC and FPCC was evaluated by means of TEM imaging. The chitosan-coated PLGA NP (PCC) presented spherical-shaped particles which were uniformly dispersed in the TEM grid (Fig. 2a, b). The conjugation of FA did increase the overall particle size; however, it retained the original shape of the particles. The particles were observed to be smooth and consistent with the DLS analysis.

### In Vitro Drug Release

**Fig. 3:**
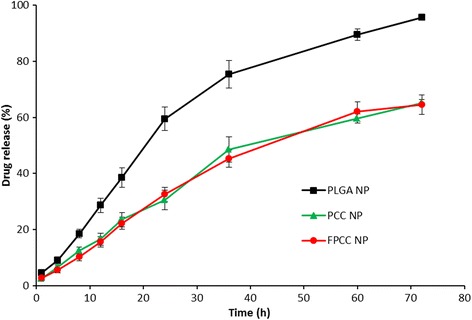
In vitro release kinetics of carboplatin-loaded chitosan-coated PLGA nanoparticles (PCC) and folic acid-conjugated chitosan-coated PLGA nanoparticles (FPCC). The release study was carried out in phosphate buffered saline (PBS) at 37 °C

The drug release was carried out using dialysis method. The release study was carried out in phosphate buffered saline (pH 7.4) in order to simulate physiological conditions. ICP-MS method was used to determine the amount of drug released. As shown in Fig. 3, CRB from PCC and FPCC released significantly slower compared to that from PLGA NP. It should be noted that nearly 60 % of drug released from PLGA NP at the end of 24 h while only 35 % of drug released from PCC and FPCC nanosystems. The slower release of drug from PCC and FPCC was mainly attributed to the presence of protective chitosan layer that might increase the path length of drug. It should be noted that the presence of FA did not alter the release rate of the drug throughout the study period [27, 28].

### Cellular Uptake Efficiency

**Fig. 4:**
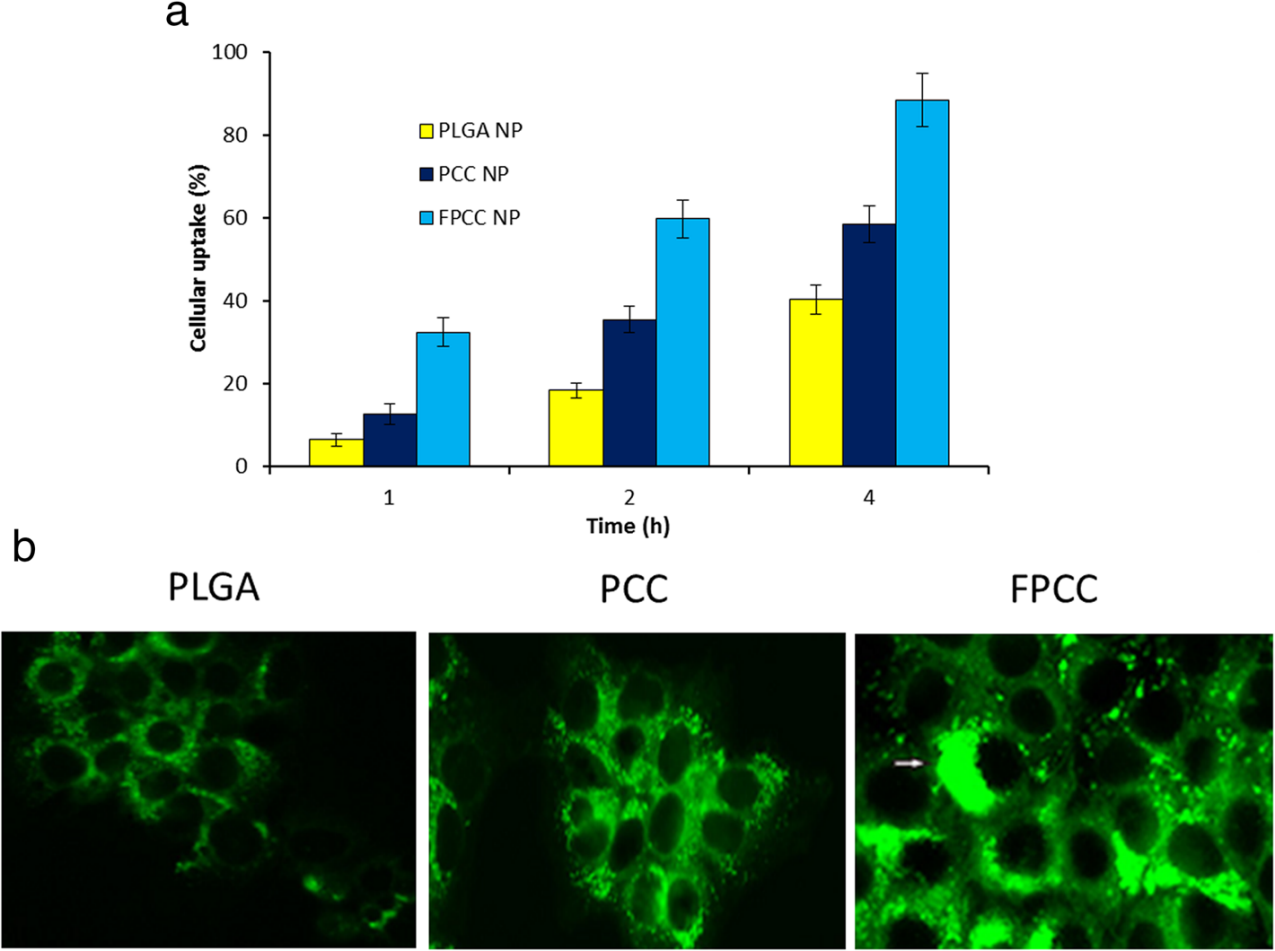
Cellular uptake analysis in cervical cancer cell (HeLa). **a** Quantitative cellular uptake analysis by fluorescence analysis and (**b**) qualitative cellular uptake in HeLa cells using confocal laser scanning microscope (CLSM). Drug was replaced with coumarin-6 for the fluorescence experiment. Quantitative cellular uptake was performed at different time points while qualitative experiment was performed after 2 h incubation

In order to exhibit the high cytotoxic effect, the intracellular concentration of drug is very important which in turn depends on the cellular uptake efficiency of nanoparticles. The cellular uptake of PLGA, PCC, and FPCC was evaluated in HeLa cells using courmarin-6 as a fluorescent probe. The study was conducted at different incubation time points. From the result, it can be clearly understood that surface morphology has a great impact on the cellular internalization capacity. One thing which is common in all the carriers is that a typical time-dependent cellular uptake was seen. PCC has higher cellular uptake than that of PLGA NP while FPCC has highest cellular uptake compared to any nanoparticles in the study (Fig. 4a). It can be safely assumed that the positively charged nanoparticle could be preferably internalized than anionic PLGA NP [29]. Evidently, enhanced cellular uptake of FPCC was attributed to the presence of FA on the surface of nanoparticles. It could be expected that enhanced cellular uptake of FPCC was due to interaction of FA with the FRs-α which is overexpressed on the HeLa and promoted higher delivery. The percent accumulation of FPCC in HeLa cells was remarkably greater than other systems. These results indicated that FPCC had a specific affinity for the cancerous, HeLa cells owing to ligand-receptor (FA-FR-α) recognition.

### Qualitative Cellular Uptake

Consistent with the quantitative cellular uptake, FPCC showed a good internalization efficiency compared to that of PCC and PLGA NP. The enhanced internalization of FPCC was evident from the bright green fluorescence of cells (Fig. 4b). This difference could be a direct consequence of surface modifications with FA while PCC uptake was greater than that of PLGA as CS coating changes the negative surface charge to a positive one. It has been reported that the internalization of cationic CS particles appears to occur predominantly by adsorptive endocytosis.

### In Vitro Cytotoxicity Assay

**Fig. 5:**
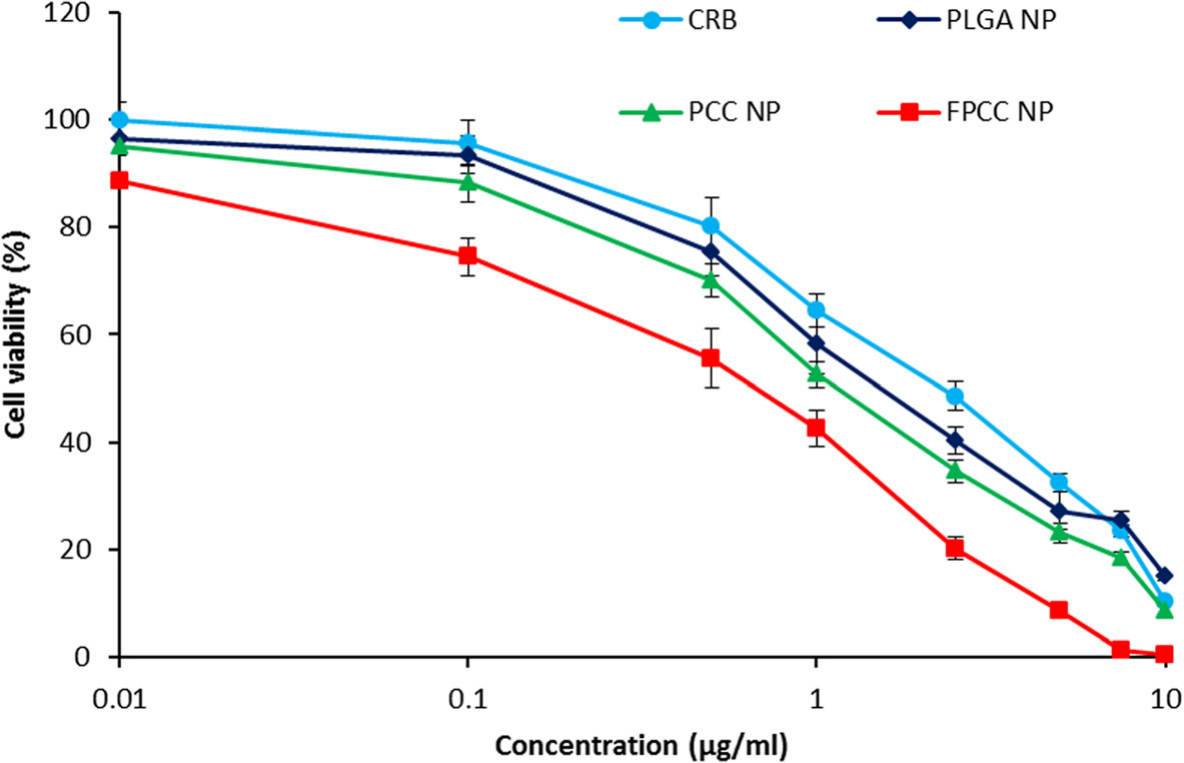
In vitro cytotoxicity analysis of CRB, PLGA NP, PCC, and FPCC in human cervical cancer, HeLa cells. The cells were incubated with formulations for 24 h and assayed by MTT protocol

To estimate the effectiveness of free CRB, PLGA NP, PCC, and FPCC on killing the HeLa cancer cells, MTT assay was performed. It can be clearly seen from the Fig. 5 that all the formulations exhibited a clear dose-dependent cytotoxicity in HeLa cancer cells. Especially, a clear trend in cytotoxicity was observed. As seen, PCC was more effective than PLGA NP while FPCC showed a superior anticancer effect after 24 h of incubation. The cytotoxicity potential of different formulations was consistent with the cellular uptake efficiency of particles. For example, PLGA NP has better penetrability than that of free drug while cationic chitosan-coated formulation has better internalization that plain PLGA NP. This could be due to the presence of cationic charge on the surface. Importantly, FPCC showed superior cytotoxic effect than any other formulations. The IC_50_ (concentration of the drug required to kill 50 % of the cells) value of FPCC was 0.65 μg/ml while it was 1.08, 1.56, and 2.35 μg/ml for PCC, PLGA NP, and free CRB, respectively. The selective uptake of FPCC and enhanced cytotoxicity in HeLa cells might improve the chemotherapeutic efficacy of carboplatin.

### Apoptosis Assay

**Fig. 6:**
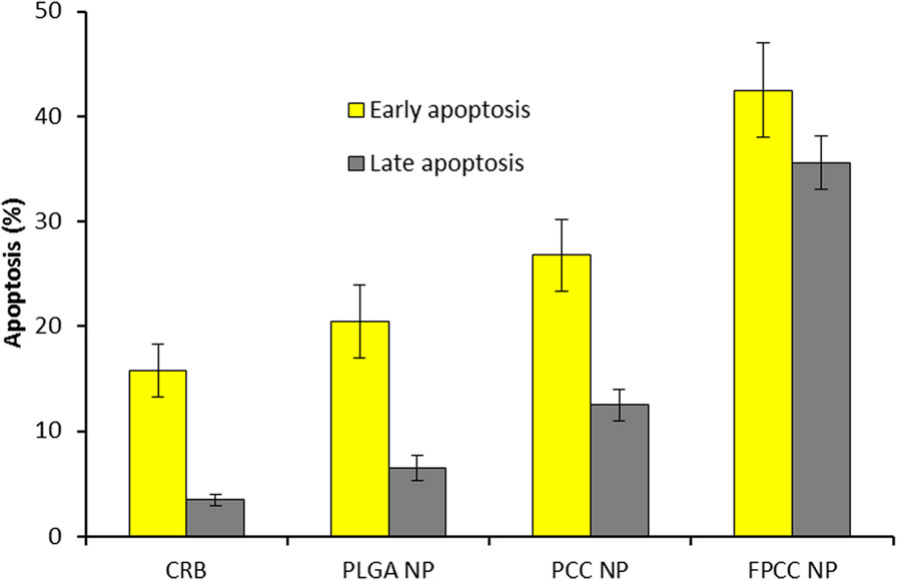
Annexin and 7-AAD staining-based apoptosis assay. Apoptosis assay carried out using fluorescent-activated cell sorting (FACS). The apoptosis was divided into early and late apoptosis. The cell was treated with 1 μg/ml equivalent of drug concentration

In the present study, apoptosis assay was performed using Annexin-V/17-AAD staining protocol in HeLa cancer cells. All the formulations induced a cell apoptosis according to their potency (Fig. 6). Significant differences in apoptosis were observed between free CRB, PLGA NP, PCC, and FPCC. Consistent with the cytotoxicity assay, FPCC induced higher fraction of early as well as late apoptosis cells. Especially, FPCC induced nearly 45 % of early apoptosis cells and more than 35 % in late apoptosis. The superior cytotoxic effect of FPCC could be attributed due to the higher internalization of FPCC inside the cells (due to the surface modification of folic acid) and continuous exposure and sustained release of the drug for a prolonged period of time at the site of action. Generally, apoptosis of cells is a normal physiological process which plays a pivotal role on the development of tissue organisms, morphological changes such as cytoplasm shrinkage, chromatin condensation, DNA fragmentation, and apoptotic body formations. Based on the result, it can be easily interpreted that folate conjugation to the nanoparticle has a great impact on the cervical cancer cell survival and could be advantageous to the cancer treatment.

## Conclusions

In summary, a unique folic acid-conjugated chitosan-coated PLGA nanoparticle was prepared for the selective delivery of carboplatin to the cervical cancer cells. The particles were nanosized and spherical shaped with size less than <200 nm. The presence of protective chitosan layer controlled the overall release rate of CRB from PCC and FPCC. FPCC displayed a higher cellular uptake capacity in HeLa cells than compared to non-targeted nanoparticles. Selective uptake of FPCC was due to interaction of FA with the FRs-α which is overexpressed on the HeLa and promoted active targeting. These results indicated that FPCC had a specific affinity for the cancerous, HeLa cells owing to ligand-receptor (FA-FR-α) recognition. Consistently, FPCC showed superior cytotoxic effect than any other formulations. The IC_50_ (concentration of the drug required to kill 50 % of the cells) value of FPCC was 0.65 μg/ml while it was 1.08, 1.56, and 2.35 μg/ml for PCC, PLGA NP, and free CRB, respectively. Consistent with the cytotoxicity assay, FPCC induced higher fraction of early as well as late apoptosis cells. Especially, FPCC induced nearly 45 % of early apoptosis cells and more than 35 % in late apoptosis. Therefore, we propose that folate-conjugated nanoparticles might have potential applications in cervical cancer therapy.
